# Nonribosomal Peptide Synthetase Specific Genome Amplification Using Rolling Circle Amplification for Targeted Gene Sequencing

**DOI:** 10.3390/ijms25105089

**Published:** 2024-05-07

**Authors:** Yoshiko Okamura, Masahiro Suemitsu, Takato Ishikawa, Hirokazu Takahashi

**Affiliations:** 1 Graduate School of Integrated Sciences for Life, Hiroshima University, Hiroshima 739-8530, Japan; m240883@hiroshima-u.ac.jp (T.I.); ziphiro@hiroshima-u.ac.jp (H.T.); 2 Graduate School of Advanced Science of Matter, Hiroshima University, Hiroshima 739-8530, Japan; su.waka.d@gmail.com

**Keywords:** rolling circle amplification, specific genome amplification, nonribosomal peptide synthetase, metagenome, viable but non-culturable (VBNC) bacteria

## Abstract

Next-generation sequencing has transformed the acquisition of vast amounts of genomic information, including the rapid identification of target gene sequences in metagenomic databases. However, dominant species can sometimes hinder the detection of rare bacterial species. Therefore, a highly sensitive amplification technique that can selectively amplify bacterial genomes containing target genes of interest was developed in this study. The rolling circle amplification (RCA) method can initiate amplification from a single locus using a specific single primer to amplify a specific whole genome. A mixed cell suspension was prepared using *Pseudomonas fluorescens* ATCC17400 (targeting nonribosomal peptide synthetase [NRPS]) and *Escherichia coli* (non-target), and a specific primer designed for the *NRPS* was used for the RCA reaction. The resulting RCA product (RCP) amplified only the *Pseudomonas* genome. The *NRPS* was successfully amplified using RCP as a template from even five cells, indicating that the single-priming RCA technique can specifically enrich the target genome using gene-specific primers. Ultimately, this specific genome RCA technique was applied to metagenomes extracted from sponge-associated bacteria, and *NRPS* sequences were successfully obtained from an unknown sponge-associated bacterium. Therefore, this method could be effective for accessing species-specific sequences of *NRPS* in unknown bacteria, including viable but non-culturable bacteria.

## 1. Introduction

A wide variety of microorganisms have been isolated from the environment and the enzymes and metabolites produced by them have been utilized. However, more than 99% of the microorganisms in the environment are difficult to cultivate, and the useful genes possessed by these microorganisms cannot be obtained using conventional culture-dependent methods, leaving a large amount of unused genetic resources [1,2]. To identify unknown useful genes, it is effective to construct and screen a metagenomic library. In general, there are two types of screenings using metagenomic libraries: sequence screening based on gene homology and functional screening based on enzyme activity [3,4]. In the former case, knowledge of the biosynthetic pathways of complex skeletal compounds, such as Erdacin, Fasamycin A, and Onnamide, has been reported [5,6,7]. In the latter case, when a positive clone is obtained based on the assay, the enzyme (or biologically active compound), its biochemical properties, and its biosynthetic genes can be simultaneously obtained. Hydrolytic enzyme esterases, environmental pollutant-degrading enzymes (extradiol dioxygenases), antibacterial *N*-acyltyrosine, and vibroferrin have been isolated from metagenomic libraries and reported [8,9,10]. We also constructed a metagenomic library from sponge-associated bacteria and reported a novel *GDSL* esterase gene out of 26,496 clones [11]. Notably, the plasmid or fosmid retains the full-length gene or operon in each report, which is essential for the functional expression of the translation product. If they were fragmented by restriction enzymes or arbitrarily cleaved by DNA sharing, functional expression in *Escherichia coli* would not be possible. Bioactive compounds, polyketides, and NRPS are produced by modular enzymes, and their biosynthetic genes form a large gene cluster [12]. Therefore, they were divided into several clones for library construction. Furthermore, the positivity rate of library screening is an issue; esterase isolated from the deep-sea sediment metagenome was screened from one hit out of 73,000 clones [8]. In some cases, only a few positive clones were obtained from 10^4^–10^6^ clones [13]. Thus, screening using metagenomic libraries is limited in obtaining genes with extremely low abundance in the environment.

Next-generation sequencing (NGS) has enabled the analysis of large amounts of genomic information. As a result of progress in sequence-based big data analysis, it is possible to obtain target gene sequences from an accumulated metagenome database. However, if the holder of a useful gene is a rare bacterial species, it may be masked by a large amount of dominant species information. Metagenome-assembled genomes (MAGs) have become feasible using NGS, and although there has been some success, a high (target) population abundance relative to the rest of the microbial community may result in poor recovery [14]. For example, there are 10^10^–10^11^ bacteria or 6000–50,000 bacterial species in 1 g of soil; not only can the entire genome be determined, but fragments of genomic information from various species are recovered as poor MAGs [15]. Recently, single-cell genomics has been reported, in which bacteria are individualized by single-cell sorting and whole-genome amplification using the multiple displacement amplification (MDA) method to secure the amount of DNA necessary for sequence analysis for use as NGS samples. This method has made it possible to obtain the individual genome sequences of even rare bacterial species [16,17]. In particular, the development of a single-cell genome analysis method using droplets has shown the possibility of solving the problems of conventional single-cell genome analysis methods, such as reduced sorting efficiency and amplification bias caused by non-cellular particles in environmental samples [18,19]. In addition, the single-cell amplified genome in the gel bead method can proceed to more than 10^5^ single cells in a tube and acquire more than 10^3^ draft genome information at a time [20,21]. Thus, the metagenomic approach in single-cell genomics has solved an obscure problem in rare species, achieved high throughput and selectivity, and revealed not only taxonomic information but also their functional genes. However, there is scope for improvement in acquisition efficiency since this approach results in whole genome amplification even in non-target single cells, and various applied analyses are required to utilize the target useful genes. In addition, high-throughput technology can be achieved by introducing sophisticated machinery, such as automated pipettors and microfluidic machines. The expense associated with this method prevents most laboratories from adopting it. Furthermore, if only certain functional genes, such as enzyme genes, are targeted, it is necessary to retain only the genome specific to the target.

Although metagenome library construction and screening have been performed to access viable but non-culturable (VBNC) bacterial genes, it is time-consuming and requires huge manpower or efficient, positive screening techniques [4]. NGS (especially long-read sequences) has opened a way to easily access VBNC resources. Moreover, sequencing fees have recently decreased to affordable prices. However, in metagenome research, huge reads are required to cover the whole genome sequence; therefore, sequencing fees are not affordable. If only the gene of interest can be enriched, increasing research costs can be avoided. Typically, to amplify the gene of interest, PCR has been often used, and universal primers have been proposed. It is very conventional and easy to access, but its amplifiable length is approximately 10 kb, which may not be applicable to unknown species.

For single-cell isolation, we developed RNase-assisted rolling circle amplification (RHa-RCA) to label specific mRNAs with fluorescence and successfully recognized specific bacteria with 30-mer nucleotides [22]. We considered that if only the target bacterial genome could be amplified by RCA with the specific primer, the PCR product of the target gene could be obtained, and a unique sequence of the PCR product could be obtained in a conventional way. Consequently, a probe for RHa-RCA can be designed based on a unique sequence from the PCR product, and fluorescence-labeled cells can be isolated. Therefore, in this study, a targeted genome enrichment method using rolling circle amplification (RCA) with targeted unique sequence primers was developed, which amplifies only a few bacterial genomes carrying the target gene, and a model experiment showed its feasibility.

## 2. Results

### 2.1. RCA Products with Single Primer (Single-Priming RCA)

**Figure 1:**
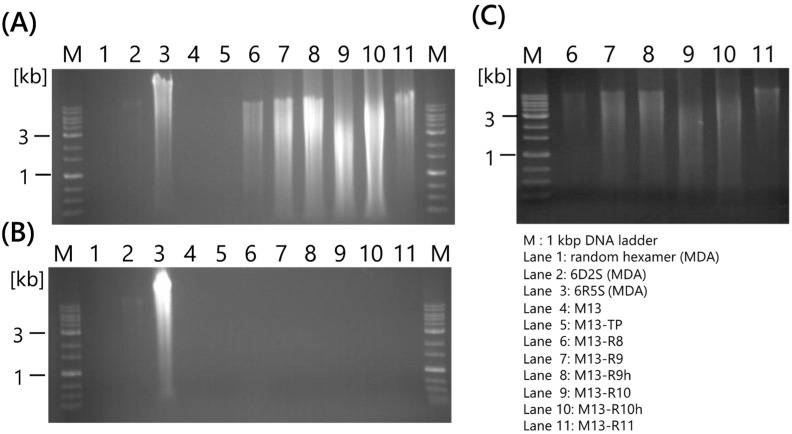
Investigation of optimal modifications in RCA primers. (**A**) Plasmid pUC19 was used as DNA template. (**B**) No-template controls. (**C**) *Eco*RI digestion of RCA products of (**A**). Random hexamers (lanes 1–3) were used for MDA. The RCA primers were designed within the M13 primer region. MDA, multiple displacement amplification; RCA, rolling circle amplification.

Phi 29 DNA polymerase has 3′-5′ exonuclease activity, with high proofreading activity and efficient strand displacement activities. Unmodified DNA primers are unsuitable for exonuclease activity; thus, thiophosphate-linkage DNA random hexamers and RNA random hexamers were used to amplify DNA using multiplied primed rolling circle amplification (MPRCA) [23,24]. Therefore, primer modification was evaluated first. Normal DNA primers, such as random 6-mer and M13, and RNA primers were designed (Table 1). As shown in a previous study, normal DNA primers did not amplify DNA using MDA and RCA (Figure 1A, Lane 1 and 4). For the thiophosphate-linked DNA primers, 6D2S and 6R5S showed the MDA product (Figure 1A, Lane 2 and 3). However, it was also observed in the no-template control (Figure 1B, Lane 2 and 3). Therefore, contaminated DNA might be in water used as a no-template control. Thiophosphate-linked M13-TP could not amplify DNA, suggesting that modification by a single thiophosphate was not sufficient to inhibit 3′-5′ exonuclease activity (Figure 1, lane 5). However, all RNA primers successfully amplified single-stranded DNA (ssDNA) because RCA products (RCPs) were not digested by *Eco*RI (Figure 1C). Moreover, no RCPs were observed, as shown in Figure 1B, lane 6–11. MPRCA amplifies contaminated DNA derived from primer dimers or airbones [25], suggesting that single-priming RCA is reliable for specific amplification.

### 2.2. Specific Amplification of pUC19 with Non-Target through Single-Priming RCA

RCP can be visualized using electrophoresis; however, the gel images show smears of highly polymerized DNA (Figure 1A). Therefore, the RCP was evaluated using PCR with specific primers for the target template DNA. Plasmid pUC19 (1 pg; 3.4 × 10^5^ copies) was mixed with *E. coli* JM109 (10^5^ cells) and used as a template for RCA. RCA primers targeting the β-lactamase gene (*bla*) were designed using RNA bases (Table 1). To confirm the RCP content, *bla*-specific primers or *E. coli*-specific primer sets were used for PCR (Table 2).

**Figure 2:**
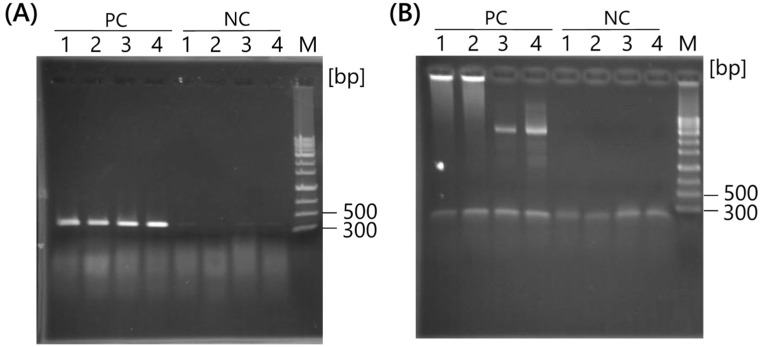
Plasmid-specific amplification by RCA and confirmation via PCR with specific primer sets. (**A**) PCR with *bla* primer set; (**B**) PCR with *E. coli* primer set. Plasmid pUC19 (1 pg; 3.4 × 10^5^ copies) was mixed with Escherichia coli JM109 (10^5^ cells) and used as a template for RCA. RCA primers (lanes 1–4) were designed within the *bla* region. PC: Template positive control; NC: no-template control. Lane M: 100 bp DNA marker; lane 1: bla10h; lane 2: bla10; lane 3: bla11; lane 4: bla12. RCA, rolling circle amplification.

One microliter of RCP was used as the PCR template and amplified with a *bla*-specific primer set or *E. coli*-specific primer set. The results showed that PCR products (387 bp) for the *bla* were confirmed in the RCPs using 10–12 mer RCA primers (Figure 2A), whereas *E. coli*-specific primer sets could not amplify DNA (585 bp) except for non-specific fragments (Figure 2B), indicating that approximately 10^5^ copies were insufficient as a PCR template. Based on the PCR results, pUC19 was amplified by RNA primers specific to *bla* with strand displacement activity, and RCP contained highly tandem repeats of pUC19, which supplied sufficient copies for the PCR template.

### 2.3. Design of NRPS-Specific RCA Primers for Pseudomonas fluorescens ATCC17400

**Figure 3:**
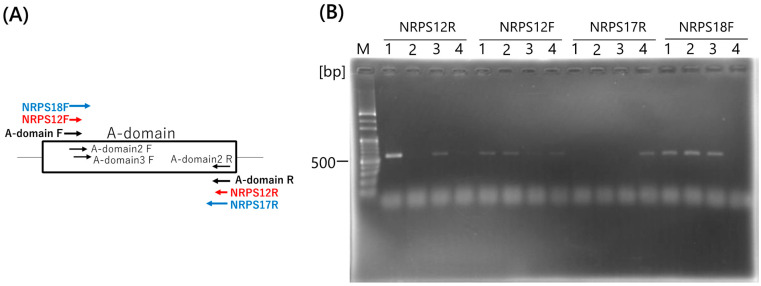
(**A**) Locations of degenerate primers (black) and RCA primers (red and blue) and (**B**) specific genome amplification by RCA and its confirmation through PCR using *Pseudomonas* genus-specific primer sets. *Pseudomonas fluorescens* ATCC17400 cells were prepared in the range of 10^4^–10 for the DNA template. Lane M: 100 bp DNA marker; lane 1: 10^4^ cells; lane 2: 10^3^ cells; lane 3: 10^2^ cells; lane 4: 10 cells. RCA, rolling circle amplification.

The bacterial genome is circular; therefore, single-priming RCA is applicable for whole-genome amplification. Our target NRPS is a modular enzyme that contains highly conserved domains; therefore, universal PCR primers for detection and sequence analysis have been reported [26]. We have found that *P. fluorescens* ATCC17400 amplified using A-domain conserved primers. Therefore, the sequence of the PCR product was determined to design NRPS-specific RCA primers. The sequence indicated in Figure S1 showed 99% homology with the nonribosomal peptide synthetase of *P. proteolytica* (accession No. WP_169910121). NRPS-specific RCA primers for *P. fluorescens* ATCC17400 were designed within the conserved regions shown in Figure 3A; the primer sequences are listed in Table 1. The Tm value of the RCA primer specific to the *NRPS* was higher than that of the primer specific to the *bla*, even at the same length (12-mer); however, the primer length was changed to increase specificity and accuracy (Table 1). To confirm genome amplification, PCR was performed using a *Pseudomonas*-specific primer set. PCR products (617 bp) were obtained using all RCA primers specific to the A-domain of NRPS, even from 10 cells (Figure 3B). This result indicated that circular DNA at the genome-scale can be applied to single-priming RCA, and sufficient copies of the PCR template can be provided using single-priming RCA. Moreover, the Tm values could be increased to at least 50 °C. Although RCA is an isothermal reaction at 30–37˚C, all RCA primers showed specific amplification (Figure 3B).

### 2.4. Specific Whole Genome Amplification in the Mixed Bacterial Flora

**Figure 4:**
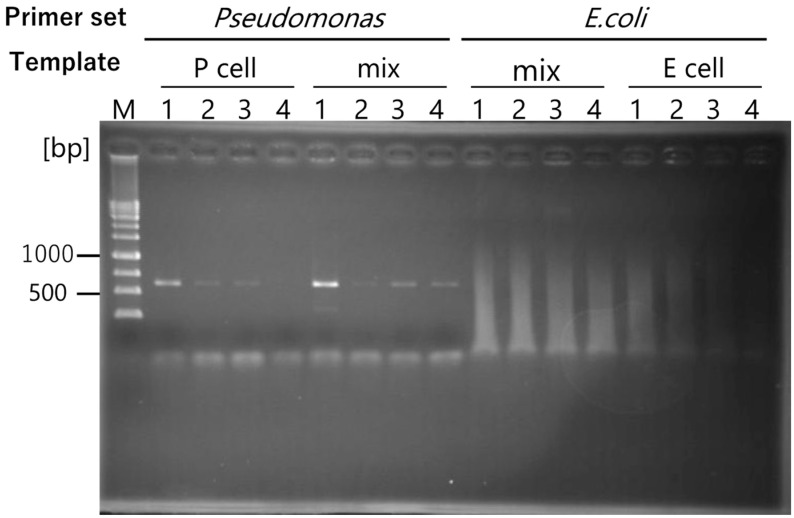
*Pseudomonas*-specific genome amplification by NRPS12R primer and confirmation via PCR with *Pseudomonas*-specific and *E. coli*-specific primer sets. The DNA template was prepared by mixing the same cell numbers of *P. fluorescens* ATCC17400 and *E. coli* JM109. P-cell: *Pseudomonas* only; E-cell: E coli only. M: 100 bp DNA marker; lane 1: 10^4^ cells; lane 2: 10^3^ cells; lane 3: 10^2^ cells; lane 4: 10 cells.

To confirm accurate, specific whole-genome amplification, the same cell numbers of *P. fluorescens* ATCC17400 and *E. coli* JM109 were mixed and used as template DNA. After single-priming amplification with NRPS-specific RCA primers, target and non-target genes were discriminated by PCR using *Pseudomonas*-specific primers and *E. coli*-specific primer sets. Even under mixed cell conditions, only a *Pseudomonas*-specific fragment (617 bp) was observed (Figure 4). In contrast, *E. coli*-specific fragments (585 bp) were not observed (Figure 4), indicating that NRPS-specific RCA primers can specifically discriminate the *Pseudomonas* genome, even from 10 cells. Additionally, when there were 10 cells/µL of mixed bacteria, five cells in the reaction tube corresponded to *Pseudomonas* cells. This result indicates that a specific genome amplification method with a targeted gene-specific RCA primer was established and that it is sensitive and accurate.

### 2.5. Application of the NRPS-Specific RCA to Unknown Environmental Bacteria

**Figure 5:**
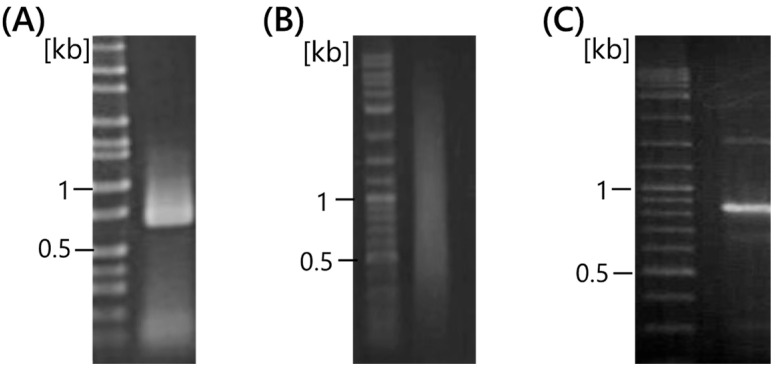
*NRPS* (A-domain) amplification using (**A**) *Pseudomonas fluorescens* ATCC17400 genome and (**A**,**B**) sponge-associated bacterial metagenome. (**A**,**B**) employed the degenerate A-domain primers from a previous report [26]. (**C**) Semi-nested PCR using A-domain 2F&2R primers for the initial PCR and A-domain 3 F & 2R primers for the second PCR.

To evaluate the versatility of NRPS-specific RCA, we focused on sponge-associated bacteria because they are often reported to include producers of bioactive compounds. To confirm the existence of the NRPS, the published universal degenerate primer set (Table 3) was used for PCR, and the primer set was applicable to *P. fluorescens* ATCC17400 and succeeded in obtaining the desired PCR product (Figure 5A). However, no PCR products were obtained from metagenomic DNA extracted from sponge-associated bacteria (Figure 5B). RCP obtained using NRPS12F was also subjected to PCR with the A-domain primer set; however, the PCR product was not observed. Universal degenerate PCR primers for NRPS were designed based on the conserved core regions A3 and A7 of NRPS adenylation A-domains [26]. The forward primer was designed based on the amino acid residue AYXXYTSG with a degeneracy of 32. This degeneracy may not be sufficient to cover the wide diversity of sponge-associated bacterial taxa. Therefore, we reconsidered the primer position at A3 and identified the following residue: YTSG(S,T)TG. The reverse primer also had a low degeneracy; therefore, the degeneracy of 256 was changed according to the same position (Figure 3A). Moreover, for semi-nested PCR, residues TG and XPKG were selected from the A3 domain to increase accuracy. The newly designed degenerate primers, named A-domain 2F, 3F and 2R, are shown in Table 3.

Then, we performed an initial PCR with the A-domain 2 primer set and size-corrected, but a smear band was obtained, and semi-nested PCR was attempted to obtain an accurate fragment. Semi-nested PCR succeeded in obtaining a clear PCR product of 700 bp from the A-domain from both RCP and the metagenome DNA without RCA (Figure 5C). NRPS12F was designed based on the YTSG residues, the most conserved residues, which was helpful for amplifying the rare bacterial genome.

Finally, the PCR product of the A-domain from sponge-associated bacteria was sequenced. The sequences of 45 clones were determined, and 35 clones showed homology with the NRPS of *P. proteokytica* (accession No. WP_169876809.1, and WP_169910121.1) with 97% identity and 98% similarity, NRPS of unclassified *Pseudomonas* (accession No. WP_169391138.1) with 97% identity and 98% similarity, and the amino acid adenylation domain of *P. versuta* (accession No. WP_125878558.1) with 60% identity and 69% similarity. Another 10 clones were out of the target. Notably, the obtained nucleotide sequences were queried in the genome database (BLASTN), and low homology with a high e-value (2 × 10^−29^) was obtained (Table S1). This clone was probably an unknown species, with the closest species being *Streptomyces* (26%). These results suggested that target-specific genome amplification can reveal species-specific sequences in hidden species.

## 3. Discussion

The RCA technique requires only a single primer and amplifies the circular DNA. Therefore, a specific primer sequence is required to enrich the specific genes of interest. This study clearly indicated that a single-priming RCA could amplify the whole genome up to sufficient copies for PCR from five cells (Figure 4). We focused on the *NRPS* gene; however, target gene-specific RCA can be applied to any gene that can provide highly conserved residues or nucleotide sequences with Tm values greater than 10 °C. Therefore, this method is suitable for use with environmental and uncultivable microbial DNA. 

The sequence of specific PCR products can suggest new primers for specific RCA to obtain genomic information in more detail. However, because RCP is ssDNA, the enriched genome cannot be used for sequencing. So far, we have attempted to add single or multiple antisense primers during specific RCA or 2nd RCA to generate double-stranded DNA (dsDNA). As a result, small circular DNA, such as pUC19, can be converted to dsDNA. Concerning the whole-genome sequencing of rare species of bacteria, the development of only this method is not enough. Fortunately, we have developed a direct mRNA labeling method, RHa-RCA-FISH [22]. Most biosynthetic gene clusters of bioactive compounds are silent [27]; therefore, DNA sequence-based screening would fail to express proteins and compounds. mRNAs are evidence of gene expression; thus, mRNA sequence-based screening is much better for acquiring the producer and their genome information. If a specific 30-mer could be obtained, a padlock probe for RHa-RCA could be designed, and fluorescence-labeled cells could be obtained using flow cytometry. Subsequently, its genome sequence could be determined. The next method for whole-genome sequencing after the isolation of bacterial cells will be reported in a future paper. As this method was established, our strategy (enrichment—fluorescence-labeling—single-cell isolation—single-cell genomics) took one step closer to the goal.

## 4. Materials and Methods

### 4.1. Plasmid, Bacterial Strains, and Culture Conditions

Plasmid pUC19 was used as a small circular DNA model. *P. fluorescens* ATCC17400 (GenBank: JENC 00000000.1) was used as a model bacterium harboring the NRPS, as the A-domain of NRPS was confirmed using PCR with conserved degenerate primers [26] and sequencing. *Pseudomonas fluorescens* ATCC17400 was grown in the Lept medium described by Boogerd and de Vrind [28] at 26 °C. The medium contained (per liter of deionized water) the following: 0.5 g of yeast extract, 0.5 g of Bacto Casamino Acids (BD Biosciences, San Jose, CA, USA), 5 mM D(+)-glucose, 10 mM HEPES (N-2-hydroxyethylpiperazine-N′-2-ethanesulfonic acid), pH 7.5, 0.48 mM CaC1_2_, 0.83 mM MgSO_4_, 3.7 µM FeCl_3_, and 1 mL of trace element solution. The trace element solution contained (per liter of deionized water) the following: 10 mg of CuSO_4_ 5H_2_O, 44 mg of ZnSO_4_ 7H_2_O, 20 mg of CoCl_2_ 6H_2_O, and 13 mg of Na_2_MoO_4_ 2H_2_O. *E. coli* JM109 cells were grown in LB medium at 37 °C. 

### 4.2. Single-Priming RCA

The cell suspensions and pUC19 were prepared via serial dilution, respectively, and 1 µL of the suspension was mixed with 5 × RPA buffer (20 mM Tris-acetate pH 7.5, 50 mM potassium acetate, 10 mM magnesium acetate, 20 mM (NH_4_)_2_SO_4_) and 200 µM RCA primer (Table 1) in a final volume of 10 µL. Primer annealing was facilitated by incubation at 95 °C for 1 min followed by ramping to 30 °C at 0.3 °C/s. After annealing, 10 µL of a reaction mixture containing 35 mM Tris-HCl (pH 7.5), 50 mM KCl, 14 mM MgCl_2_, 10 mM (NH_4_)_2_SO_4_, 4 mM dithiothreitol, 1 mM dNTPs, 40 U of Phi29 DNA polymerase (New England Biolabs, Japan), and 0.002 U of inorganic pyrophosphatase (Takara Bio, Shiga, Japan) was added to the tube (total 20 µL), which was incubated for 16 h at 30 °C, followed by incubation for 10 min at 65 °C to inactivate the enzyme. To reduce the high viscosity of the amplification products, they were transferred to 1.5 mL centrifuge tubes and mixed with 180 µL dDW by pipetting, followed by vortex mixing. The diluted products were digested with *Eco*RI (Takara Bio) to recognize single-stranded DNA.

**Table 1:** List of RCA primers used in this study.

Name	Sequence	Length	Modification	Tm (°C)
random 6-mer	5′-NNNNNN-3′	6 mer	normal	
6D2S [29]	5′-NNNN*N*N-3′	6 mer	*thiophosphate	
6R5S [29]	5′-rN*rN*rN*rN*rN*rN-3′	6 mer	RNA, *thiophosphate	
M13	5′-AGATAGGTGCGTGTTAG-3′	17 mer	normal	48.1
M13-TP	5′-AGATAGGTGCGTGTTA*G-3′	17 mer	DNA,*thiophosphate	39.5
M13-R8	5′-ACAAGCCC-3′	8 mer	RNA	2.5
M13-R9	5′-CAGACAAGC-3′	9 mer	RNA	9.0
M13-R9h	5′-ACAAGCCCG-3′	9 mer	RNA	13.5
M13-R10	5′-CAGACAAGCC-3′	10 mer	RNA	18.3
M13-R10h	5′-GACAAGCCCG-3′	10 mer	RNA	22.4
M13-R11	5′-CAGACAAGCCC-3′	11 mer	RNA	25.9
bla10h	5′-AUACCGCGCC-3′	10 mer	RNA	22.4
bla10	5′-GGAUAAUACC-3′	10 mer	RNA	10.1
bla11	5′-GAUAAUACCGC-3′	11 mer	RNA	18.5
bla12	5′-GGAUAAUACCGC-3′	12 mer	RNA	25.5
NRPS12R	5′-CCGGUGCGGUAC-3′	12 mer	RNA	35.7
NRPS12F	5′-CUACACGUCGGG-3′	12 mer	RNA	32.3
NRPS17R	5′-CCGGUGCGGUACAACCG-3′	17 mer	RNA	50.4
NRPS18F	5′-CUACACGUCGGGCUCCAC-3′	18 mer	RNA	51.0

The asterisk (*) indicates a thiophosphate-linked nucleotide. RCA, rolling circle amplification.

### 4.3. PCR

To confirm specific RCA amplification, PCR was performed using a specific primer set (Table 2). TaKaRa LA Taq (Takara Bio) was used with 1 µL of RCP as a template and 20 pmol of each primer in a total volume of 20 µL. The thermal cycling conditions were as follows: pre-denaturation at 95 °C for 1 min, followed by 25 cycles of denaturation at 95 °C for 30 s, annealing at 55 °C for 30 s, and extension at 72 °C for 30 s. When using the degenerate primer sets shown in Table 3, ramping PCR was employed, and the thermal cycling conditions were as follows: pre-denaturation at 98 °C for 10 s, followed by 40 cycles of denaturation at 94 °C for 30 s, ramping to 59 °C at 0.3 °C /s, annealing for 45 s, and extension at 72 °C for 1 min, and an additional extension at 72 °C for 5 min. The PCR products were confirmed by electrophoresis on a 1.5% agarose gel.

**Table 2:** List of polymerase chain reaction (PCR) primers used in this study.

PCR Primer Name		Sequence	Reference
*bla* primer set	F	5′-TTGCCGGGAAGCTAGAGTAA-3′	This study
	R	5′-GCTATGTGGCGCGGTATTAT-3′	
*Escherichia coli* primer set	F	5′-GACCTCGGTTTAGTTCACAGA -3′	[30]
	R	5′-CACACGCTGACGCTGACCA-3′	
*Pseudomonas* genus primer set	F	5′-TTGCCGGGAAGCTAGAGTAA-3′	[29]
	R	5′-GCTATGTGGCGCGGTATTAT-3′	

**Table 3:** List of degenerate primers used in this study.

Degenerate Primer Name		Sequence	Amino Acids Sequence	Degeneracy
A-domain primer set [26]	F	5′-GCSTACSTSATCTACACSTCSGG-3′	AYXXYTSG	32
	R	5′-CCAGGTCVCCSGTSCGGTA-3′	YRYGDL	12
A-domain 2F primer	F	5′-TAYACNWSSGGYACYACYGG-3′	YTSG(S,T)TG	512
A-domain 2R primer	R	5′-CAGRTCRCCNGTNCKRTA-3′	Y(K,R)TGDL	256
A-domain 3F primer	F	5′-ACSGGYRWNCCNAARGG-3′	TGXPKG	512

### 4.4. DNA Sequencing and Homology Analysis

The PCR product was cloned into the pGEM-T-easy vector pGEM-T-easy Vector System (PROMEGA). After confirmation of the gene insertion, the plasmid extracted from the clone was sequenced by Eurofins Genomics Co., Ltd. (Tokyo, Japan). The chromatograms of the DNA sequences were quality checked and trimmed using SnapGene^®^ Viewer 6.1.1. The sequences were analyzed using the National Center for Biotechnology Information (NCBI) Basic Local Alignment Search Tool (BLAST); accessed on 30 March 2024. BLASTN was used to search the host genome. BLASTX was used against a non-redundant protein database to confirm the coding amino acid sequences.

### 4.5. Preparation of Sponge-Associated Bacterial Cells and DNA Extraction

Marine sponges *Halichondria panicea* were collected from the coastal beach of Hinomisaki, Shimane, Japan, and used to isolate the associated bacteria. The bacteria were collected, and genomic DNA was extracted as previously described [11]. The sponge was homogenized using a mortar and pestle in TNE buffer (10 mM Tris, 3.5% NaCl, 50 mM EDTA, pH 7.5), and the sponge tissue was removed via filtration through a 100 µm mesh. The nuclei were removed from the filtrate via centrifugation at 700× *g* for 5 min. Bacterial cells were harvested from the resulting supernatant via centrifugation at 7000× *g* for 10 min and washed three times with TNE buffer. DNA was extracted from the bacteria using the following method: collected cells (approximately 1.2 g) were resuspended in 10 mL TE buffer (10 mM Tris, 1 mM EDTA, pH 8.0) containing 500 mg lysozyme and incubated for 2 h at 37 °C. Then, 1.4 mg Proteinase K and 10 mg RNase) were added and the mixture, which was incubated for an additional 1 h. To this mixture, 3.2 mL of 10% sodium dodecyl sulfate was added and incubated at 37 °C overnight. Further, 11 mL of 5 M NaCl and 8 mL of cetyl trimethylammonium bromide (CTAB)/NaCl solution (10% CTAB in 0.7 M NaCl) were added to the mixture and incubated at 65 °C for 30 min. DNA was separated from the insoluble material via centrifugation at 10,000× *g* for 10 min, resulting in the enrichment of DNA in the supernatant. The DNA was purified using two standard phenol–chloroform extractions. The extracted DNA (metagenome DNA) was purified twice using cesium chloride density-gradient centrifugation at 55,000× *g* for 16 h. The resulting DNA was dialyzed for 24 h to remove the cesium chloride. The resulting genomic DNA was used as RCA and PCR templates.

### 4.6. Semi-Nested PCR

Semi-nested PCR was performed using two-step PCR. The first PCR was performed using A-domain 2F and 2R primers and 10 ng of metagenome DNA as a template. The second PCR was performed using A-domain 3F and 2R primers and 1 µL of the first PCR product as a template. The thermal cycling conditions when using degenerate primers are described in Section 4.3.

## 5. Conclusions

A method for gene-specific genome enrichment was established in this study. Single-priming RCA with gene-specific primer can successfully amplify specific genomes, even from 10 cells. The rare bacterial genome can be amplified to a sufficient copy number for PCR, and subsequent DNA sequencing provides specific DNA sequences derived from unknown bacteria or VBNC bacteria. This result suggested that this method could be effective for accessing species-specific sequences of *NRPS* genes in unknown bacteria, including VBNC bacteria, at affordable prices.

## 6. Patents

This research is connected to Japanese patent number 6112652, which was issued on 24 March 2017.

## Data Availability

The data that support the findings of this study are available from the corresponding author upon reasonable request.

## Supplementary Materials

The following supporting information can be downloaded at: https://www.mdpi.com/article/10.3390/ijms25105089/s1.
