# Supplementary material for: Nonribosomal Peptide Synthetase Specific Genome Amplification Using Rolling Circle Amplification for Targeted Gene Sequencing
Source: Int J Mol Sci. 2024 May 7;25(10):5089. doi: 10.3390/ijms25105089 (PMC11121399; doi:10.3390/ijms25105089)
Supplement: Supplementary file 1 [file ijms-25-05089-s001.zip › ijms-2967075-supplementary.pdf]

Nonribosomal Peptide Synthetase (NRPS) Specific Genome Amplification using Rolling Circle Amplification for Targeted Gene Sequencing

Yoshiko Okamura[1, 2]*, Masahiro Suemitsu[2], Takato Ishikawa[1], Hirokazu Takahashi[1]

[1]Graduate School of Integrated Sciences for Life, Hiroshima University

[2]Graduate School of Advanced Science of Matter, Hiroshima University

*Correspondence: okamuray@hiroshima-u.ac.jp; Tel.: +81-82-424-4583

Supplemental Figures and Tables

Supplemental Figures and Tables.

1	*TGCGTACGTGATCTACACGTCGGG*CTCCACCGGCAACCCCCAAGGGTGTG
51	GCGATTGCTCATCGCAATGTGCTGGCGCTGATCCATTGGTCGCAGCAAGT
101	CTATAGTCGTGAGGACATCCAGGGCGTGCTGGCCTCCACTTCGGTGTGCT
151	TCGACCTGTCGGTCTGGGAAATCTTCGTCACCCTGGCCAATGGCGGCTCA
201	ATCGTCCTTGCACGCAATGCCCTGGAGCTGCCGGAATTGCTGGGGCGGGA
251	ACAGGTCCGCCTGATAAATACCGTTCCATCGGCGATCAATGCCCTGCAGC
301	GAGCCGGACAAATCCCTGACAGCGTGCGCATCATCAACCTGGCCGGCGAA
351	CCGCTGAAGCAGTCGCTGGTGGACAGCCTCTACCAACAAGCCACGATCAA
401	GCACATCTTCGATCTTTACGGCCCGTCCGAAGACACCACCTACTCTACCT
451	GGACTCGCCGCGAGGGCGGCGGGCAGGCCAATATTGGCCGGCCATTGCAC
501	AACACTCAAAGCTATCTGCTCGATGCCGACCTGCAACCGGTACCAACCGG
551	GGTGGCGGCCGAACTGCACTTGGCAGGTGCAGGGATTACCCGAGGCTATC
601	TCGGGCGCGCAGCCATGACTGCGGAAAAATATGTACCCAACCCGTTCTCC
651	ACTACGGGTGAACGGTTG*TACCGCACCGGGGACCTGG*

Figure S1. Nucleotide sequence of PCR product for the A-domain of NRPS in *Pseudomonas fluorescens* ATCC17400.

PCR primer sequences were indicated by Italic letters. RCA primers were designed within the under lined regions.

Supplemental Table 1. Homology analysis of the nucleotide sequences from sponge-associated bacteria.

	BLASTN	Accession #	Query cover	E-value		BLASTX	Accession #	Query cover
AD5	Streptomyces sp. CB01881	CP026498.1	26%	2E-29		amino acid adenylation domain-containing protein [Pseudomonas versuta]	WP_125878558.1	96%
AD13	Pseudomonas sp ADAK20	CP052858.1	98%	0.0		non-ribosomal peptide synthetase [Pseudomonas proteolytica]	WP_169876809.1	97%
AD23	Pseudomonas sp ADAK20	CP052858.1	92%	0.0		non-ribosomal peptide synthetase [Pseudomonas proteolytica]	WP_169876809.1	89%
AD32	Pseudomonas sp ADAK20	CP052858.1	98%	0.0		non-ribosomal peptide synthetase [Pseudomonas proteolytica]	WP_169876809.1	97%
AD37	Pseudomonas sp ADAK20	CP052858.1	98%	0.0		non-ribosomal peptide synthetase [**unclassified Pseudomonas**]	WP_169391138.1	97%
AD38	Pseudomonas sp ADAK20	CP052858.1	98%	0.0		non-ribosomal peptide synthetase [Pseudomonas proteolytica]	WP_169876809.1	97%
AD40	Pseudomonas sp ADAK20	CP052858.1	98%	0.0		non-ribosomal peptide synthetase [Pseudomonas proteolytica]	WP_169876809.1	97%
AD45	Pseudomonas sp ADAK20	CP052858.1	98%	0.0		non-ribosomal peptide synthetase [Pseudomonas proteolytica]	WP_169910121.1	94%
AD47	Pseudomonas sp ADAK20	CP052858.1	93%	0.0		non-ribosomal peptide synthetase [**unclassified Pseudomonas**]	WP_169391138.1	90%
AD48	Pseudomonas sp ADAK20	CP052858.1	98%	0.0		non-ribosomal peptide synthetase [Pseudomonas proteolytica]	WP_169876809.1	97%
AD50	Pseudomonas sp ADAK20	CP052858.1	93%	0.0		non-ribosomal peptide synthetase [Pseudomonas proteolytica]	WP_169876809.1	90%
AD80	Pseudomonas sp ADAK20	CP052858.1	98%	0.0		non-ribosomal peptide synthetase [Pseudomonas proteolytica]	WP_169876809.1	97%
AD130	Pseudomonas sp ADAK20	CP052858.1	94%	0.0		non-ribosomal peptide synthetase [Pseudomonas proteolytica]	WP_169890519.1	91%
AD210	Pseudomonas sp ADAK20	CP052858.1	91%	0.0		non-ribosomal peptide synthetase [Pseudomonas proteolytica]	WP_169876809.1	89%
AD300	Pseudomonas sp ADAK20	CP052858.1	88%	0.0		non-ribosomal peptide synthetase [Pseudomonas proteolytica]	WP_169890519.1	96%
AD370	Pseudomonas sp ADAK20	CP052858.1	93%	0.0		non-ribosomal peptide synthetase [Pseudomonas proteolytica]	WP_169876809.1	90%
AD380	Pseudomonas sp ADAK20	CP052858.1	99%	0.0		non-ribosomal peptide synthetase [unclassified Pseudomonas]	WP_169391138.1	97%
AD400	Pseudomonas sp ADAK20	CP052858.1	100%	0.0		non-ribosomal peptide synthetase [Pseudomonas proteolytica]	WP_169876809.1	97%
AD450	Pseudomonas sp ADAK20	CP052858.1	98%	0.0		non-ribosomal peptide synthetase [Pseudomonas proteolytica]	WP_169876809.1	97%
AD480	Pseudomonas sp ADAK20	CP052858.1	92%	0.0		non-ribosomal peptide synthetase [unclassified Pseudomonas]	WP_169391138.1	87%
AD470	Pseudomonas sp ADAK20	CP052858.1	99%	0.0		non-ribosomal peptide synthetase [unclassified Pseudomonas]	WP_169391138.1	93%
AD460	Pseudomonas sp ADAK20	CP052858.1	100%	2E-155		non-ribosomal peptide synthetase [Pseudomonas proteolytica]	WP_169890519.1	92%
AD390	Pseudomonas sp ADAK20	CP052858.1	98%	0.0		non-ribosomal peptide synthetase [Pseudomonas proteolytica]	WP_169876809.1	96%
AD300	Pseudomonas sp ADAK20	CP052858.1	98%	0.0		non-ribosomal peptide synthetase [Pseudomonas proteolytica]	WP_169876809.1	96%
AD290	Pseudomonas sp ADAK20	CP052858.1	93%	0.0		non-ribosomal peptide synthetase [Pseudomonas proteolytica]	WP_169910121.1	96%
AD220	Pseudomonas sp ADAK20	CP052858.1	98%	0.0		non-ribosomal peptide synthetase [Pseudomonas proteolytica]	WP_169876809.1	96%
AD70	Pseudomonas sp ADAK20	CP052858.1	98%	0.0		non-ribosomal peptide synthetase [Pseudomonas proteolytica]	WP_169876809.1	96%
AD60	Pseudomonas sp ADAK20	CP052858.1	100%	0.0		non-ribosomal peptide synthetase [Pseudomonas proteolytica]	WP_169876809.1	96%
AD46	Pseudomonas sp ADAK20	CP052858.1	98%	0.0		non-ribosomal peptide synthetase [Pseudomonas proteolytica]	WP_169876809.1	96%
AD31	Pseudomonas sp ADAK20	CP052858.1	100%	0.0		non-ribosomal peptide synthetase [Pseudomonas proteolytica]	WP_169876809.1	96%
AD22	Pseudomonas sp ADAK20	CP052858.1	98%	0.0		non-ribosomal peptide synthetase [Pseudomonas proteolytica]	WP_169876809.1	96%
AD21	Pseudomonas sp ADAK20	CP052858.1	98%	0.0		non-ribosomal peptide synthetase [Pseudomonas proteolytica]	WP_169910121.1	96%
AD16	Pseudomonas sp ADAK20	CP052858.1	98%	0.0		non-ribosomal peptide synthetase [Pseudomonas proteolytica]	WP_169876809.1	96%
AD14	Pseudomonas sp ADAK20	CP052858.1	98%	0.0		non-ribosomal peptide synthetase [Pseudomonas proteolytica]	WP_169876809.1	96%
AD6	Pseudomonas sp ADAK20	CP052858.1	96%	2E-155		non-ribosomal peptide synthetase [Pseudomonas proteolytica]	WP_169876809.1	96%

Query sequences for Supplemental Table 1

>MG_AD5

ACGGGTAAGCCGAAGGGGGTGATGCTCACTCATCTCGCGTTGATCAACCTGC

TGGACTGGTTCCAGCACGATGACGGCCTCATGTATGGTGGTCGGGTTCTGCA

GAAGACGCCATTCTCCTTTGACGTCTCCGTCAGGGAATTTCTCTGGCCGCTG

ACCCAAGGCTCGATGCTCGTCGTTCTTCCACCCGAAGACCATCGAGATCCGG

CCCGTCTGCGGGCGGCCATTACGAAGTTCGGCATCACGACCCTGCACTTCGT

GCCATCGATGCTGCAGGCATTTATGGTTACAGGCGGCTTCGCCGGGACCTCG

GAGCTACGTCGCATCTTTGTCGGCGGGGAGGCGACGCCGGCCATGCTAGCAC

GCCAGGTCCGCGATCAGACTCCGGCCGCCGTCTACAATCAGTACGGCCCGAC

TGAAACGGCGATCGATGTAACGCGTCACCGGATCGTCGAGAGTGATCTAGGA

CGCATTCCGATTGGTCGTCCGATCTCGAACACGCGGGTTTATGTTCTTGATGC

TGAGCTTCAGCCTGTTCCTGTGGGTGTTCCGGGCGAGCTTTACATAGCGGGT

CATGGTCTTGCGCGGGGTTATCTGAACCGTCCGGGTCTGACGTCGGAGCGGT

TCGTGGCGTGTCCGTTCGGCCAAGCGGGCGAGCGGATGTATCGCACCGGCG

ACCTGA

>MG_AD13

ACCGGTGTGCCGAAGGGTGTGGTGATTGCTCATCGCAATGTGCTGGCGCTGA

TCTATTGGTCGCAACAAGTCTATAGTCGTGAGGACATCCAGGGCGTGCTGGC

CTCCACTTCGGTGTGCTTCGACCTGTCGGTCTGGGAAATCTTCGTCACCCTG

GCCAATGGCGGCTCAATCGTCCTTGCACGCAATGCCCTGGAGCTGCCGGAAT

TGCTGGGGCGGGAACAGGTCCGCCTGATAAATACCGTTCCATCGGCGATCAA

TGCCCTGCAGCGAGCCGGACAAATCCCTGACGGCGTGCGCATCATCAACCTG

GCCGGCGAACCGCTGAAGCAGTCGCTGGTGGACAGCCTCTACCAACAAGCC

ACGATCAAGCACATCTTCGATCTTTACGGCCCGTCCGAAGACACCACCTACT

CTACCTGGACTCGCCGCGAGGGCGGCGGGCGGGCCAATATTGGCCGGCCATT

GCACAACACTCAAAGCTATCTGCTCGATGCCGACCTGCAACCGGTACCAACC

GGGGTGGCGGCCGAACTGCACTTGGCAGGTGCAGGGATTACCCGAGGCTAT

CTCGGGCGCGCAGCCATGACTGCGGAAAAATATGTACCCAACCCGTTCTCCA

CTACGGGTGAACGGTTGTACCGCACCGGCGACCTGA

>MG_AD23

ACCGGCGAACCCAAGGGTGTGGCGATTGCTCATCGCAATGTGCTGGCGCTGA

TCCATTGGTCGCAGCAAGTCTATAGTCGTGAGGACATCCAGGGCGTGCTGGC

CTCCACTTCGGTGTGCTTCGACCTGTCGGTCTGGGAAATCTTCGTCACCCTG

GCCAATGGCGGCTCAATCGTCCTTGCACGCAATGCCCTGGAGCTGCCGGAAT

TGCTGGGGCGGGAACAGGTCCGCCTGATAAATACCGTTCCATCGGCGATCAA

TGCCCTGCAGCGAGCCGGACAAATCCCTGACGGCGTGCGCATCATCAACCTG

GCCGGCGAACCGCTGAAGCAGTCGCTGGTGGACAGCCTCTACCAACAAGCC

ACGATCAAGCACATCTTCGATCTTTACGGCCCGTCCGAAGACACCACCTACC

CTACCTAGACTCGCCGCGAGGGCGGCGGGCGGGCCAATATTGGCCGGCCATT

GCACAACACTCAAAGCTATCTGCTCGATGCCGACCTGCAACCGGTACCAACC

GGGGTGGCGGCCGAACTGCACTTGGCAGGTGCAGGGATTACCCGAGGCTAT

CTCGGGCGCGCAGCCATGACTGCGGAAAAATATGTACCCAACCCGTTCTCCA

CTACGGGTGAGCCTAAAGGGATTTATGGTGGAGCATGATACCGAACCCGGCG

ACCTGA

>MG_AD32

ACGGGCGTTCCAAAGGGTGTGGCGATTGCTCATCGCAATGTGCTGGCGCTGA

TCCATTGGTCGCAGCAAGTCTATAGTCGTGAGGACATCCAGGGCGTGCTGGC

CTCCACTTCGGTGTGCTTCGACCTGTCGGTCTGGGAAATCTTCGTCACCCTG

GCCAATGGCGGCTCAATCGTCCTTGCACGCAATGCCCTGGAGCCGCCGGAAT

TGCTGGGGCGGGAACAGGTCCGCCTGATAAATACCGTTCCATCGGCGATCAA

TGCCCTGCAGCGAGCCGGACAAATCCCTGACGGCGTGCGCATCATCAACCTG

GCCGGCGAACCGCTGAAGCAGTCGCTGGTGGACAGCCTCTACCAACAAGCC

ACGATCAAGCACATCTTCGATCTTTACGGCCCGTCCGAAGACACCACCTACT

CTACCTGGACTCGCCGCGAGGGCGGCGGGCGGGCCAATATTGGCCGGCCATT

GCACAACACTCAAAGCTATCTGCTCGATGCCGACCTGCAACCGGTACCAACC

GGGGTGGCGGCCGAACTGCACTTGGCAGGTGCAGGGATTACCCGAGGCTAT

CTCGGGCGCGCAGCCATGACTGCGGAAAAATATGTACCCAACCCGTTCTCCA

CTACGGGTGAACGGTTGTACCGCACCGGCGATCTGA

>MG_AD37

ACGGGCGATCCTAAGGGTGTGGCGATTGCTCATCGCAATGTGCTGGCGCTGA

TCCATTGGTCGCGGCAAGTCTATAGTCGTGAGGACATCCAGGGCGTGCTGGC

CTCCACTTCGGTGTGCTTCGACCTGTCGGTCTGGGAAATCTTCGTCACCCTG

GCCAATGGCGGCTCAATCGTCCTTGCACGCAATGCCCTGGAGCTGCCGGAAT

TGCTGGGGCGGGGACAGGTCCGCCTGATAAATACCGTTCCATCGGCGATCAA

TGCCCTGCAGCGAGCCGGACAAATCCCTGACGGCGTGCGCATCATCAACCTG

GCCGGCGAACCGCTGAAGCAGTCGCTGGTGGACAGCCTCTACCAACAAGCC

ACGATCAAGCACATCTTCGATCTTTACGGCCCGTCCGAAGACACCACCTACT

CTACCTGGACTCGCCGCGAGGGCGGCGGGCGGGCCAATATTGGCCGGCCATT

GCACAACACTCAAAGCTATCTGCTCGATGCCGACCTGCAACCGGTACCAACC

GGGGTGGCGGCCGAACTGCACTTGGCAGGTGCAGGGATTACCCGAGGCTAT

CTCGGGCGCGCAGCCATGACTGCGGAAAAATATGTACCCAACCCGTTCTCCA

CTACGGGTGAACGGTTGTACCGAACGGGTGACCTGAGA

>MG_AD38

ACGGGTGTCCCGAAGGGTGTGGCGATTGCTCATCGCAATGTGCTGGCGCTGA

TCCATTGGTCGCAGCAAGTCTATAGTCGTGAGGACATCCAGGGCGTGCTGGC

CTCCACTTCGGTGTGCTTCGACCTGTCGGTCTGGGAAATCTTCGTCACCCTG

GCCAATGGCGGCTCAATCGTCCTTGCACGCAATGCCCTGGAGCTGCCGGAAT

TGCTGGGGCGGGAACAGGTCCGCCTGATAAATACCGTTCCATCGGCGATCAA

TGCCCTGCAGCGAGCCGGACAAATCCCTGACGGCGTGCGCATCATCAACCTG

GCCGGCGAACCGCTGAAGCAGTCGCTGGTGGACAGCCTCTACCAACAAGCC

ACGATCAAGCACATCTTCGATCTTTACGGCCCGTCCGAAGACACCACCTACT

CTACCTGGACTCGCCGCGAGGGCGGCGGGCGGGCCAATATTGGCCGGCCATT

GCACAACACTCAAAGCTATCTGCTCGATGCCGACCTGCAACCGGTACCAACC

GGGGTGGCGGCCGAACTGCACTTGGCAGGTGCAGGGATTACCCGAGGCTAT

CTCGGGCGCGCAGCCATGACTGCGGAAAAATATGTACCCAACCCGTTCTCCA

CTACGGGTGAACGGTTGTACCGGACAGGCGACCTGA

>MG_AD40

ACGGGTGTTCCGAAGGGTGTGGTGATTGCTCATCGCAATGTGCTGGCGCTGA

TCCATTGGTCGCAGCAAGTCTATAGTCGTGAGGACATCCAGGGCGTGCTGGC

CTCCACTTCGGTGTGCTTCGACCTGTCGGTCTGGGAAATCTTCGTCACCCTG

GCCAATGGCGGCTCAATCGTCCTTGCACGCAATGCCCTGGAGCTGCCGGAAT

TGCTGGGGCGGGAACAGGTCCGCCTGATAAATACCGTTCCATCGGCGATCAA

TGCCCTGCAGCGAGCCGGACAAATCCCTGACGGCGTGCGCATCATCAACCTG

GCCGGCGAACCGCTGAAGCAGTCGCTGGTGGACAGCCTCTACCAACAAGCC

ACGATCAAGCACATCTTCGATCTTTACGGCCCGTCCGAAGACACCACCTACT

CTACCTGGACTCGCCGCGAGGGCGGCGGGCGGGCCAATATTGGCCGGCCATT

GCACAACACTCAAAGCTATCTGCTCGATGCCGACCTGCAACCGGTACCAACC

GGGGTGGCGGCCGAACTGCACTTGGCAGGTGCAGGGATTACCCGAGGCTAT

CTCGGGCGCGCAGCCATGACTGCGGAAAAATATGTACCCAACCCGTTCTCCA

CTACGGGTGAACGGTTGTACCGCACCGGCGACCTGA

>MG_AD45

ACGGGCGAGCCAAAGGGTGTGGCGATTGCTCATCGCAATGTGCTGGCGCTG

ATCCATTGGTCGCAGCAAGTCTATAGTCGTGAGGACATCCAGGGCGTGCTGG

CCTCCACTTCGGTGTGCTTCGACCTGTCGGTCTGGGAAATCTTCGTCACCCTG

GCCAATGGCGGCTCAATCGTCCTTGCACGCAATGCCCTGGAGCTGCCGGAAT

TGCTGGGGCGGGAACAGGTCCGCCTGATAAATACCGTTCCATCGGCGATCAA

TGCCCTGCAGCGAGCCGGACAAATCCCTGACAGCGTGCGCATCATCAACCTG

GCCGGCGAACCGCTGAAGCAGTCGCTGGTGGACAGCCTCTACCAACAAGCC

ACGATCAAGCACATCTTCGATCTTTACGGCCCGTCCGAAGACACCACCTACT

CTACCTGGACTCGCCGCGAGGGCGGCGGGCAGGCCAATATTGGCCGGCCATT

GCACAACACTCAAAGCTATCTGCTCGATGCCGACCTGCAACCGGTACCAACC

GGGGTGGCGGCCGAACTGCACTTGGCAGGTGCAGGGATTACCCGAGGCTAT

CTCGGGCGCGCAGCCATGACTGCGGAAAAATATGTACCCAACCCGTTCTCCA

CTACGGGTGAACGGTTGTACCGTACCGGCGACCTGA

>MG_AD47

ACCGGCGATCCCAAGGGTGTGGTGATTGCTCATCGCAATGTGCTGGCGCTGA

TCCATTGGTCGCAGCAAGTCTATAGTCGTGAGGACATCCAGGGCGTGCTGGC

CTCCACTTCGGTGTGCTTCGACCTGTCGGTCTGGGAAATCTTCGTCACCCTG

GCCAATGGCGGCTCAATCGTCCTTGCACGCAATGCCCTGGAGCTGCCGGAAT

TGCTGGGGCGGGAACAGGTCCGCCTGATAAATACCGTTCCATCGGCGATCAA

TGCCCTGCAGCGAGCCGGACAAATCCCTGACGGCGTGCGCATCATCAACCTG

GCCGGCGAACCGCTGAAGCAGTCGCTGGTGGACAGCCTCTACCAACAAGCC

ACGATCAAGCACATCTTCGATCTTTACGGCCCGTCCGAAGACACCACCTACT

CTACCTGGACTCGCCGCGAGGGCGGCGGGCGGGCCAATATTGGCCGGCCATT

GCGCAACACTCAGAGCTATCTGCTCGATGCCGACCTGCAACCGGTACCAACC

GGGGTGGCGGCCGAACTGCACTTGGCAGGTGCAGGGATTACCCGAGGCTAT

CTCGGGCGCGCAGCCATGACTGCGGAAAAATATGTACCCAACCCGTTCTCCA

CTACGGGCGAGCCTAANGGATTTATGGTGGAGCATGATACAGCACCGGCGAC

CTGA

>MG_AD48

ACGGGCGAACCTAAGGGTGTGGCGATTGCTCATCGCAATGTGCTGGCGCTGA

TCCATTGGTCGCAGCAAGTCTATAGTCGTGAGGACATCCAGGGCGTGCTGGC

CTCCACTTCGGTGTGCTTCGACCTGTCGGTCTGGGAAATCTTCGTCACCCTG

GCCAATGGCGGCTCAATCGTCCTTGCACGCAATGCCCTGGAGCCGCCGGAAT

TGCTGGGGCGGGAACAGGTCCGCCTGATAAATACCGTTCCATCGGCGATCAA

TGCCCTGCAGCGAGCCGGACAAATCCCTGACGGCGTGCGCATCATCAACCTG

GCCGGCGAACCGCTGAAGCAGTCGCTGGTGGACAGCCTCTACCAACAAGCC

ACGATCAAGCACATCTTCGATCTTTACGGCCCGTCCGAAGACACCACCTACT

CTACCTGGACTCGCCGCGAGGGCGGCGGGCGGGCCAATATTGGCCGGCCATT

GCACAACACTCAAAGCTATCTGCTCGATGCCGACCTGCAACCGGTACCAACC

GGGGTGGCGGCCGAACTGCACTTGGCAGGTGCAGGGATTACCCGAGGCTAT

CTCGGGCGCGCAGCCATGACTGCGGAAAAATATGTACCCAACCCGTTCTCCA

CTACGGGTGAACGGTTGTACCGCACAGGCGACCTGA

>MG_AD50

ACCGGCGTGCCCAAGGGTGTGGTGATTGCTCATCGCAATGTGCTGGCGCTGA

TCCATTGGTCGCAGCAAGTCTACAGTCGTGAGGACATCCAGGGCGTGCTGGC

CTCCACTTCGGTGTGCTTCGACCTGTCGGTCTGGGAAATCTTCGTCACCCTG

GCCAATGGCGGCTCAATCGTCCTTGCACGCAATGCCCTGGAGCTGCCGGAAT

TGCTGGGGCGGGAACAGGTCCGCCTGATAAATACCGTTCCATCGGCGATCAA

TGCCCTGCAGCGAGCCGGACAAATCCCTGACGGCGTGCGCATCATCAACCTG

GCCGGCGAACCGCTGAAGCAGTCGCTGGTGGACAGCCTCTACCAACAAGCC

ACGATCAAGCACATCTTCGATCTTTACGGCCCGTCCGAAGACACCACCTACT

CTACCTGGACTCGCCGCGAGGGCGGCGGGCGGGCCAATATTGGCCGGCCATT

GCACAACACTCAAAGCTATCTGCTCGATGCCGACCTGCAACCGGTACCAACC

GGGGTGGCGGCCGAACTGCGCTTGGCAGGTGCAGGGATTACCCGAGGCTAT

CTCGGGCGCGCAGCCATGACTGCGGAAAAATATGTACCCAGCCCGTTCTCCA

CTACGGGCGTGCCGAAAGGGTTTATCGTACCGGACCGGCGACCTGA

>MG_AD80

ACGGGTGAACCAAAGGGTGTGGCGATTGCTCATCGCAATGTGCTGGCGCTGA

TCCATTGGTCGCAGCAAGTCTATAGTCGTGAGGACATCCAGGGCGTGCTGGC

CTCCACTTCGGTGTGCTTCGACCTGTCGGTCTGGGAAATCTTCGTCACCCTG

GCCAATGGCGGCTCAATCGTCCTTGCACGCAATGCCCTGGAGCTGCCGGAAT

TGCTGGGGCGGGAACAGGTCCGCCTGATAAATACCGTTCCATCGGCGATCAG

TGCCCTGCAGCGAGCCGGACAAATCCCTGACAGCGTGCGCATCATCAACCTG

GCCGGCGAACCGCTGAAGCAGTCGCTGGTGGACGGCCTCTACCAACAAGCC

ACGATCAAGCACATCTTCGATCTTTACGGCCCGTCCGAAGACACCACCTACT

CTACCTGGACTCGCCGCGAGGGCGGCGGGCAGGCCAATATTGGCCGGCCATT

GCACAACACTCAAAGCTATCTGCTCGATGCCGACCTGCAACCGGTACCAACC

GGGGTGGCGGCCGAACTGCACTTGGCAGGTGCAGGGATTACCCGAGGCTAT

CTCGGGCGCGCAGCCATGACTGCGGAAAAATATGTACCCAACCCGTTCTCCA

CTACGGGTGAACGGTTGTACCGCACAGGCGACCTGA

>MG_AD130

ACGGGCGTACCGAAGGGTGTGGTGATTGCTCATCGCAATGTGCTGGCGCTGA

TCCATTGGTCGCAGCAAGTCTATAGTCGTGAGGACATCCAGGGCGTGCTGGC

CTCCACTTCGGTGTGCTTCGACCTGTCGGTCTGGGAAATCTTCGTCACCCTG

GCCAATGGCGGCTCAATCGTCCTTGCACGCGATGCCCTGGAGCTGCCGGAAT

TGCTGGGGCGGGAACAGGTCCGCCTGATAAATACCGTTCCATCGGCGATCAA

TGCCCTGCAGCGAGCCGGACAAATCCCTGACGGCGTGCGCATCATCAACCTG

GCCGGCGAACCGCTGAAGCAGTCGCTGGTGGACAGCCTCTACCAACAAGCC

ACGATCAAGCACATCTTCGATCTTTACGGCCCGTCCGAAGACACCACCTACT

CTACCTGGACTCGCCGCGAGGGCGGCGGGCGGGCCAATATTGGCCGGCCATT

GCACAACACTCAAAGCTACCTGCTCGATGCCGACCTGCAACCGGTACCAACC

GGGGTGGCGGCCGAACTGCACTTGGCAGGTGCAGGGATTACCCGAGGCTAT

CTCGGGCGCGCAGCCATGACTGCGGAAAAATATGTACCCAACCCGTTCTCCA

CTACGGGTGAGCCAAAGGGGTGGACGATACCGCACCGGCGACCTGA

>MG_AD210

ACGGGCGTTCCCAAGGGTGTGGCGATTGCTCATCGCAATGTGCTGGCGCTGA

TCCATTGGTCGCAGCAAGTCTATAGTCGTGAGGACATCCAGGGCGTGCTGGC

CTCCACTTCGGTGTGCTTCGACCTGTCGGTCTGGGAAATCTTCGTCACCCTG

GCCAATGGCGGCCCAATCGTCCTTGCACGCAATGCCCTGGAGCTGCCGGAAT

TGCTGGGGCGGGAACAGGTCCGCCTGATAAATACCGTTCCATCGGCGATCAA

TGCCCTGCAGCGAGCCGGACAAATCCCTGACGGCGTGCGCATCATCAACCTG

GCCGGCGAACCGCTGAAGCAGTCGCTGGTGGACAGCCTCTACCAACAAGCC

ACGATCAAGCACATCTTCGATCTTTACGGCCCGTCCGAAGACACCACCTACT

CTACCTGGACTCGCCGCGAGGGCGGCGGGCGGGCCAATATTGGCCGGCCATT

GCACAACACTCAAAGCTATCTGCTCGATGCCGACCTGCAACCGGTACCAACC

GGGGTGGCGGCCGAACTGCACTTGGCAGGTGCAGGGATTACCCGAGGCTAT

CTCGGGCGCGCAGCCATGACTGCGGAAAAATATGTACCCAACCCGTTCTCCA

CTACGGGCGTACCAAAGGGGGTCAAAGGCGGGCTTGATACCGAACTGGCGA

CCTGA

>MG_AD230

ACCGGCGTGCCGAAGGGTGTGGTGATTGCTCATCGCAATGTGCTGGCGCTGA

TCCATTGGTCGCAGCAAGTCTATAGTCGTGAGGACATCCAGGGCGTGCTGGC

CTCCACTTCGGTGTGCTTCGACCTGTCGGTCTGGGAAATCTTCGTCACCCTG

GCCAATGGCGGCTCAATCGTCCTTGCACGCAATGCCCTGGAGCTGCCGGAAT

TGCTGGGGCGGGAACAGGTCCGCCTGATAAATACCGTTCCATCGGCGATCAA

TGCCCTGCAGCGAGCCGGACAAATCCCTGACGGCGTGCGCATCATCAACCTG

GCCGGCGAACCGCTGAAGCGGTCGCTGGTGGACAGCCTCTACCAACAAGCC

ACGATCAAGCACATCTTCGATCTTTACGGCCCGTCCGAAGACACCACCTACT

CTACCTGGACTCGCCGCGAGGGCGGCGGGCGGGCCAATATTGGCCGGCCATT

GCACAACACTCAAAGCTATCTGCTCGATGCCGACCTGCAACCGGTACCAACC

GGGGTGGCGGCCGAACTGCACTTGGCAGGTGCAGGGATTACCCGAGGCTAT

CTCGGGCGCGCAGCCATGACTGCGGAAAAATATGTACCCAACCCGTTCTCCA

CTACGGGCGAGCCGAA

>MG_AD370

ACGGGCGAACCGAAGGGTGTGGTGATTGCTCATCGCAATGTGCTGGCGCTGG

TCCATTGGTCGCAGCAAGTCTATAGTCGTGAGGACATCCAGGGCGTGCTGGC

CTCCACTTCGGTGTGCTTCGACCTGTCGGTCTGGGAAATCTTCGTCACCCTG

GCCAATGGCGGCTCAATCGTCCTTGCACGCAATGCCCTGGAGCTGCCGGAAT

TGCTGGGGCGGGAACAGGTCCGCCTGATAAATACCGTTCCATCGGCGATCAA

TGCCCTGCAGCGAGCCGGACAAATCCCTGACGGCGTGCGCATCATCAACCTG

GCCGGCGAACCGCTGAAGCAGTCGCTGGTGGACAGCCTCTACCAACAAGCC

ACGATCAAGCACATCTTCGATCTTTACGGCCCGTCCGAAGACACCACCTACT

CTACCTGGACTCGCCGCGAGGGCGGCGGGCGGGCCAATATTGGCCGGCCATT

GCACAACACTCAAAGCTATCTGCTCGATGCCGACCTGCAACCGGTACCAACC

GGGGTGGCGGCCGAACTGCACTTGGCAGGTGCAGGGATTACCCGAGGCTAT

CTCGGGCGCGCAGCCATGACTGCGGAAAAATATGTACCCAACCCGTTCTCCA

CTACGGGCGAACCAAAGGGATTTATGGTGGAGCATGATACCGAACTGGCGAC

CTGA

>MG_AD380

ACGGGCGACCCAAAGGGTGTGGCGATTGCTCATCGCAATGTGCTGGCGCTGA

TCCATTGGTCGCAGCAAGTCTATAGTCGTGAGGACATCCAGGGCGTGCTGGC

CTCCACTTCGGTGTGCTTCGACCTGTCGGTCTGGGAAATCTTCGTCACCCTG

GCCAATGGCGGCTCAATCGTCCTTGCACGCAATGCCCTGGAGCTGCCGGAAT

TGCTGGGGCGGGAACAGGTCCGCCTGATAAATACCGTTCCATCAGCGATCAA

TACCCTGCAGCGAGCCGGACAAATCCCTGACAGCGTGCGCATCATCAACCTG

GCCGGCGAACCACTGAAGCAGTCGCTGGTGGACAGCCTCTACCAACAAGCC

ACGATCAAGCACATCTTCGGTCTTTACGGCCCGTCCGAAGACACCGCCTACT

CTACCTGGACTCGCCGCGAGGGCGGCGGGCAGGCCAATATTGGCCGGCCATT

GCACAACACTCAAAGCTATCTGCTCGATGCCGACCTGCAACCGGTACCAACC

GGGGTGGCGGCCGAACTGCACTTGGCAGGTGCAGGGATTACCCGAGGCTAT

CTCGGGCGCGCAGCCATGACTGCGGAAAAATATGTACCCAACCCGTTCTCCA

CTACGGGTGAACGGTTGTACCGCACCGGTGACCTGA

>MG_AD400

ACGGGTATGCCGAAGGGTGTGGTGATTGCTCATCGCAATGTGCTGGCGCTGA

TCCATTGGTCGCAGCAAGTCTATAGTCGTGAGGACATCCAGGGCGTGCTGGC

CTCCACTTCGGTGTGCTTCGACCTGTCGGTCTGGGAAATCTTCGTCACCCTG

GCCAATGGCGGCTCAATCGTCCTTGCACGCAATGCCCTGGAGCTGCCGGAAT

TGCTGGGGCGGGAGCAGGTCCGCCTGATAAATACCGTTCCATCGGCGATCAA

TGCCCTGCAGCGAGCCGGACAAATCCCTGACGGCGTGCGCATCATCAACCTG

GCCGGCGAACCGCTGAAGCAGTCGCTGGTGGATAGCCTCTACCAACAAGCC

ACGATCAAGCACATCTTCGATCTTTACGGCCCGTCCGAAGACACCACCTACT

CTACCTGGACTCGCCGCGAGGGCGGCGGGCGGGCCAATATTGGCCGGCCATT

GCACAACACTCAAAGCTATCTGCTCGATGCCGACCTGCAACCGGTACCAACC

GGGGTGGCGGCCGAACTGCACTTGGCAGGTGCAGGGATTACCCGAGGCTAT

CTCGGGCGCGCAGCCATGACTGCGGAAAAATATGTACCCAACCCGTTCTCCA

CTACGGGTGAACGGTTGTACCGTACCGGCGACCTGA

>MG_AD450

ACCGGTGTGCCTAAGGGTGTGGCGATTGCTCATCGCAATGTGCTGGCGCTGA

TCCATTGGTCGCAGCAAGTCTATAGTCGTGAGGACATCCAGGGCGTGCTGGC

CTCCACTTCGGTGTGCTTCGACCTGTCGGTCTGGGAAATCTTCGTCACCCTG

GCCAATGGCGGCTCAATCGTCCTTGCACGCAATGCCCTGGAGCTGCCGGAAT

TGCTGGGGCGGGAACAGGTCCGCCTGATAAATACCGCTCCATCGGCGATCAA

TGCCCTGCAGCGAGCCGGACAAATCCCTGACAGCGTGCGCATCATCAACCTG

GCCGGCGAACCGCTGAAGCAGTCGCTGGTGGACGGCCTCTACCAACAAGCC

ACGATCAAGCACATCTTCGATCTTTACGGCCCGTCCGAAGACACCACCTACT

CTACCTGGACTCGCCGCGAGGGCGGCGGGCAGGCCAATATTGGCCGGCCATT

GCACAACACTCAAAGCTATCTGCTCGATGCCGACCTGCAACCGGTACCAACC

GGGGTGGCGGCCGAACTGCACTTGGCAGGTGCAGGGATTACCCGAGGCTAT

CTCGGGCGCGCAGCCATGACTGCGGAAAAATATGTACCCAACCCGTTCTCCA

CTACGGGTGAACGGTTGTACAGCACCGGCGACCTGA

>MG_AD480

ACTAGTGATTACGGGCGATCCCAAGGGTGTGGTGATTGCTCATCGCAATGTGC

TGGCGCTGATCCATTGGTCGCAGCAAGTCTATAGTCGTGAGGACATCCAGGG

CGTGCTGGCCTCCACTTCGGTGTGCTTCGACCTGTCGGTCTGGGAAATCTTC

GTCACCCTGGCCAATGGCGGCTCAATCGTCCTTGCACGCAATGCCCTGGAGC

TGCCGGAATTGCTGGGGCGGGAACAGGTCCGCCTGATAAATACCGTTCCATC

GGCGATCAATGCCCTGCAGCGAGCCGGACAAATCCCTGACGGCGTGCGCATC

ATCAACCTGGCCGGCGAACCGCTGAAGCAGTCGCTGGTGGACAGCCTCTAC

CAACAAGCCACGATCAAGCACATCTTCGATCTTTACGGCCCGTCCGAAGACA

CCACCTACTCTACCTGGACTCGCCGCGAGGGCGGCGGGCGGGCCAATATTGG

CCGGCCATTGCACAACACTCAAAGCTATCTGCTCGATGCCGACCTGCAACCG

GTACCAACCGGGGTGGCGGCCGAACTGCACTTGGCAGGTGCAGGGATTACC

CGAGGCTATCTCGGGCGCGCAGCCATGACTGCGGAAAAATATGTACCCAACC

CGTCCTCCACTACGGGTGAACCGAAAGGGGAACCGTCTTGGNATTGAAATAC

CGGACCGGCGACCTGA

>MG_AD470

CAGGTCGCCTGTGCGGTACAACCGTTCACCCGTAGTGGAGAACGGGTTGGG

TACAAATTTTTCCGCAGTCATGTCTGCGCGCCCGAGATAGCCTCGGGTAATCC

CTGCACCTGCCAAGTGCAGTTCGGCCGCCACCCCGGTTGGTACCGGTTGCAG

GTCGGCATCGAGCAGATAGCTTTGAGTGTTGTGCAATGGCCGGCCAATATTG

GCCCGCCCGCCGCCCTCGCGGCGAGTCCAGGTAGAGTAGGTGGTGTCTTCGG

ACGGGCCGTAAAGATCGAAGATGTGCTTGATCGTGGCTTGTTGGTAGAGGCT

GTCCACCAGCGACTGCTTCAGCGGTTCGCCGGCCAGGTTGATGATGCGCACG

CCGTCAGGGATTTGTCCGGCTCGCTGCAGGGCATTGATCGCCGATGGAACGG

TATTTATCAGGCGGACCTGTTCCCGCCCCAGCAATTCCGGCAGCTCCAGGGC

ATTGCGTGCAAGGACGATTGAGCCGCCATTGGCCAGGGTGACGAAGATTCCC

CAGACCGACAGGTCGAAGCACACCGAAGTGGAGGCCAGCACGCCCTGGATG

TCCTCACGACTATAGACTTGCTGCGACCAATGGATCAGCGCCAGCACATTGC

GATGAGCAATCACCACACCCTTCGGGTCGCCCGTA

>MG_AD460

CAGGTCACCGGTGCGGTACAACCGTTCACCCGTAGTGGAGAACGGGTTGGG

TACATATTTTTCCGCAGTCATGGCTGCGCGCCCGAGATAGCCTCGGGTAATCC

CTGCACCTGCCAAGTGCAGTTCGGCCGCCACCCCGGTTGGTACCGGTTGCAG

GTCGGCATCGAGCAGATAGCTTTGAGTGTTGTGCAATGGCCGGCCAGTATTG

GCCTGCCCGCCGCCCTCGCGGCGAGTCCAGGTAGAGTAGGTGGTGTCTTCGG

ACGGGCCGTAAAGATCGAAGATGTGCTTGATCGTGGCTTGTTGGTAGAGGCC

GTCCACCAGCGACTGCTTCAGCGGTTCGCCGGCCAGGTTGATGATGCGCACG

CTGTCAGGGATTTGTCCGGCTCGCTGCAGGGCATTGATCGCCGATGGAACGG

TATTTATCAGGCGGACCTGTTCCCGCCCCAGCAATTCCGGCAGCTCCAGGGC

ATTGCGTGCAAGGACGATTGAGCCGCCATTGGCCAGGGTGACGAAGATTTCC

CAGACCGACAGGTCGAAGCACACCGAAGTGGAGGCCAGCACGCCCTGGATG

TCCTCACGACTATAGACTTGCTGCGACCAATGGATCAGCGCCAGCACATTGC

GATGAGCAATCGCCACACCCTTTCGGCTCGCCCGTA

>MG_AD390

CAGGTCGCCGGTTCTGTACAACCGTTCACCCGTAGTGGAGAACGGGTTGGGT

ACATATTTTTCCGCAGTCATGGCTGCGCGCCCGAGATAGCCTCGGGTAATCCC

TGCACCTGCCAAGTGCAGTTCGGCCGCCACCCCGGTTGGTACCGGTTGCAGG

TCGGCATCGAGCAGATAGCTTTGAGTGTTGTGCAATGGCCGGCCAATATTGGC

CCGCCCGCCGCCCTCGCGGCGAGTCCAGGTAGAGTAGGTGGTGTCTTCGGAC

GGGCCGTAAAGATCGAAGATGTGCTTGATCGTGGCTTGTTGGTAGAGGCTGT

CCACCAGCGACTGCTTCAGCGGTTCGCCGGCCAGGTTGATGATGCGCACGCC

GTCAGGGATTTGTCCGGCTCGCTGCAGGGCATTGATCGCCGATGGAACGGTA

TTTATCAGGCGGACCTGTTCCCGCCCCAGCAATTCCGGCGGCTCCAGGGCAT

TGCGTGCAAGGACGATTGAGCCGCCATTGGCCAGGGTGACGAAGATTTCCCA

GACCGACAGGTCGAAGCACACCGAAGTGGAGGCCAGCACGCCCTGGATGTC

CTCACGACTATAGACTTGCTGCGACCAATGGATCAGCGCCAGCACATTGCGA

TGAGCAATCGCCACACCCTTCGGAACGCCCGTA

>MG_AD300

CAGGTCGCCGGTGCGGTACAACCGTTCACCCGTAGTGGAGAACGGGTTGGG

TACATATTTTTCCGCAGTCATGGCTGCGCGCCCGAGATAGCCTCGGGTAATCC

CTGCACCTGCCAAGTGCAGTTCGGCCGCCACCCCGGTTGGTACCGGTTGCAG

GTCGGCATCGAGCAGATAGCTTTGAGTGTTGTGCAATGGCCGGCCAATATTG

GCCCGCCCGCCGCCCTCGCGGCGAGTCCAGGTAGAGTAGGTGGTGTCTTCGG

ACGGGCCGTAAAGATCGAAGATGTGCTTGATCGTGGCTTGTTGGTAGAGGCT

GTCCACCAGCGACTGCTTCAGCGGTTCGCCGGCCAGGTTGATGATGCGCACG

CCGTCAGGGATTTGTCCGGCTCGCTGCAGGGCACTGATCGCCGATGGAACGG

TATTTATCAGGCGGACCTGTTCCCGCCCCAGCAATTCCGGCGGCTCCAGGGC

ATTGCGTGCAAGGACGATTGAGCCGCCATTGGCCAGGGTGACGAAGATTTCC

CAGACCGACAGGTCGAAGCACACCGAAGTGGAGGCCAGCACGCCCTGGATG

TCCTCACGACTATAGACTTGCTGCGACCAATGGATCAGCGCCAGCACATTGC

GATGAGCAATCGCCACACCCTTAGGAACACCCGTA

>MG_AD290

CAGGTCGCCTGTACGGTATCGCGCATCTTGAGCCCTTCGGCACGCCCGTAGT

GGAGAACGGGTTGGGTACATATTTTTCCGCAGTCATGGCTGCGCGCCCGAGA

TAGCCTCGGGTAATCCCTGCACCTGCCAAGTGCAGTTCGGCCGCCACCCCGG

TTGGTACCGGTTGCAGGTCGGCATCGAGCAGATAGCTTTGAGTGTTGTGCAA

TGGCCGGCCAATATTGGCCCGCCCGCCGCCCTCGCGGCGAGTCCAGGTAGAG

TAGGTGGTGTCTTCGGACGGGCCGTAAAGATCGAAGATGTGCTTGATCGTGG

CTTGTTGGTAGGGGCTGTCCACCAGCGACTGCTTCAGCGGTTCGCCGGCCAG

GTTGATGATGCGCACGCCGTCAGGGATTTGTCCGGCTCGCTGCAGGGCATTG

ATCGCCGATGGAACGGTATTTATCAGGCGGACCTGTTCCCGCCCCAGCAATTC

CGGCGGCTCCAGGGCATTGCGTGCAAGGACGATTGAGCCGCTATTGGCCAGG

GTGACGAAGATTTCCCAGACCGACAGGTCGAAGCACACCGAAGTGGAGGCC

AGCACGCCCTGGATGTCCTCACGACTATAGACTTGCTGCGACCAATGGATCA

GCGCCAGCACATTGCGATGAGCAATCGCCACACCCTTTTGGATCGCCGGTA

>MG_AD220

CAGGTCGCCCGTTCGGTACAACCGTTCACCCGTAGTGGAGAACGGGTTGGGT

ACATATTTTTCCGCAGTCATGGCTGCGCGCCCGAGATAGCCTCGGGTAATCCC

TGCACCTGCCAAGTGCAGTTCGGCCGCCACCCCGGTTGGTACCGGTTGCAGG

TCGGCATCGAGCAGATAGCTTTGAGCGTTGTGCAATGGCCGGCCAATATTGG

CCTGCCCGCCGCCCTCGCGGCGAGTCCAGGTAGAGTAGGTGGTGTCTTCGGA

CGGGCCGTAAAGATCGAAGATGTGCTTGATCGTGGCTTGTTGGTAGAGGCCG

TCCACCAGCGACTGCTTCAGCGGTTCGCCGGCCAGGTTGATGATGCGCACGC

TGTCAGGGATTTGTCCGGCTCGCCGCAGGGCATTGATCGCCGATGGAACGGT

ATTTATCAGGCGGACCTGTTCCCGCCCCAGCAATTCCGGCAGCTCCAGGGCA

TTGCGTGCAAGGACGATTGAGCCGCCATTGGCCAGGGTGACGAAGATTTCCC

AGACCGACAGGTCGAAGCACACCGAAGTGGAGGCCAGCACGCCCTGGATGT

CCTCACGACTATAGACTTGCTGCGACCAATGGATCAGCGCCAGCACATTGCG

ATGAGCAATCGCCACACCCTTTGGCACGCCCGTA

>MG_AD70

CAGGTCGCCTGTGCGGTACAACCGTTCACCCGTAGTGGAGAACGGGTTGGG

TACATATTTTTCCGCAGTCATGGCTGCGCGCCCGAGATAGCCTCGGGTAATCC

CTGCACCTGCCAAGTGCAGTTCGGCCGCCACCCCGGTTGGTACCGGTTGCAG

GTCGGCATCGAGCAGATAGCTTTGAGTGTTGTGCAATGGCCGGCCAATATTG

GCCTGCCCGCCGCCCTCGCGGCGAGTCCAGGTAGAGTAGGTGGTGTCTTCGG

ACGGGCCGTAAAGATCGAAGACGTGCTTGATCGTGGCTTGTTGGTAGAGGCC

GTCCACCAGCGACTGCTTCAGCGGTTCGCCGGCCAGGTTGATGATGCGCACG

CTGTCAGGGATTTGTCCGGCTCGCTGCAGGGCATTGATCGCCGATGGAACGG

TATTTATCAGGCGGACCTGTTCCCGCCCCAGCAATTCCGGCAGCTCCAGGGC

ATTGCGTGCAAGGACGATTGAGCCGCCATTGGCCAGGGTGACGAAGATTTCC

CAGACCGACAGGTCGAAGCACACCGAAGTGGAGGCCAGCACGCCCTGGATG

TCCTCACGACTATAGACTTGCTGCGACCAATGGATCAGCGCCAGCACATTGC

GATGAGCAATCGCCACACCCTTGGGCACGCCGGTA

>MG_AD60

CAGGTCGCCGGTGCTGTACAACCGTTCACCCGTAGTGGAGAACGGGTTGGG

TACATATTTTTCCGCAGTCATGGCTGCGCGCCCGAGATAGCCTCGGGTAATCC

CTGCACCTGCCAAGTGCAGTTCGGCCGCCACCCCGGTTGGTACCGGTTGCAG

GTCGGCATCGAGCAGATAGCTTTGAGTGTTGTGCAATGGCCGGCCAATATTG

GCCCGCCCGCCGCCCTCGCGGCGAGTCCAGGTAGAGTAGGTGGTGTCTTCGG

ACGGGCCGTAAAGATCGAAGATGTGCTTGATCGTGGCTTGTTGGTAGAGGCT

GTCCACCAGCGACTGCTTCAGCGGTTCGCCGGCCAGGTTGATGATGCGCACG

CCGTCAGGGATTTGTCCGGCTCGCTGCAGGGCATTGATCGCCGATGGAACGG

TATTTATCAGGCGGACCTGTTCCCGCCCCAGCAATTCCGGCAGCTCCAGGGC

ATTGCGTGCAAGGACGATTGAGCCGCCATTGGCCAGGGTGACGAAGATTTCC

CAGACCGACAGGTCGAAGCACACCGAAGTGGAGGCCAGCACGCCCTGGATG

TCCTCACGACTATAGACTTGCTGCGACCAATGGGTCAGCGCCAGCACATTGC

GATGAGCAATCGCCACACCCTTAGGCTCGCCGGTA

>MG_AD46

TACGGGTGTGCNTAAGGGTGTGGCGATTGCTCATCGCAATGTGCTGGCGCTG

ATCCATTGGTCGCAGCAAGTCTATAGTCGTGAGGACATCCAGGGCGTGCTGG

CCTCCACTTCGGTGTGCTTCGGCCTGTCGGTCTGGGAAATCTTCGTCACCCTG

GCCAATGGCGGCTCAATCGTCCTTGCACGCAATGCCCTGGAGCTGCCGGAAT

TGCTGGGGCGGGAACAGGTCCGCCTGATAAATACCGTTCCATCGGCGATCAA

TGCCCTGCAGCGAGCCGGACAAATCCCTGACGGCGTGCGCATCATCAACCTG

GCCGGCGAACCGCTGAAGCAGTCGCTGGTGGACAGCCTCTACCAACAAGCC

ACGATCAAGCACATCTTCGATCTTTACGGCCCGTCCGAAGACACCACCTACT

CTACCTGGACTCGCCGCGAGGGCGGCGGGCGGGCCAATATTGGCCGGCCATT

GCACAACACTCAAAGCTATCTACTCGATGCCGACCTGCAACCGGTACCAACC

GGGGTGGCGGCCGAACTGCACTTGGCAGGTGCAGGGATTACCCGAGGCTAT

CTCGGGCGCGCAGCCATGACTGCGGAAAAATATGTACCCAACCCGCTCTCCA

CTACGGGTGAACGGTTGTACCGCACCGGTGACCTG

>MG_AD31

CAGGTCGCCTGTACGGTACAACCGTTCACCCGTAGTGGAGAACGGGTTGGGT

ACATATTTTTCCGCAGTTATGGCTGCGCGCCCGAGATAGCCTCGGGTAATCCC

TGCACCTGCCAAGTGCAGTTCGGCCGCCACCCCGGTTGGTACCGGTTGCAGG

TCGGCATCGAGCAGATAGCTTTGAGTGTTGTGCAATGGCCGGCCAATATTGGC

CCGCCCGCCGCCCTCGCGGCGAGTCCAGGTAGAGTAGGTGGTGTCTTCGGAC

GGGCCGTAAAGATCGAAGATGTGCTTGATCGTGGCTTGTTGGTAGAGGCTGT

CCACCAGCGACTGCTTCAGCGGTTCGCCGGCCAGGTTGATGATGCGCACGCC

GTCAGGGATTTGTCCGGCTCGCTGCAGGGCATTGATCGCCGATGGAACGGTA

TTTATCAGGCGGACCTGTTCCCGCCCCAGCAATTCCGGCAGTTCCAGGGCAT

TGCGTGTAAGGACGATTGAGCCGCCATTGGCCAGGGTGACGAAGATTTCCCA

GACCGACAGGTCGAAGCACACCGAAGTGGAGGCCAGCACGCCCTGGATGTC

CTCACGACTATAGACTTGCTGCGACCAATGGATCAGCGCCAGCACACTGCGA

TGAGCAATCACCACACCCTTCGGCACACCCGTA

>MG_AD22

CAGGTCGCCGGTTCGGTACAACCGTTCACCCGTAGTGGAGAACGGGTTGGG

TACATATTTTTCCGCAGTCATGGCTGCGCGCCCGAGATAGCCTCGGGTAATCC

CTGCACCTGCCAAGTGCAGTTCGGCCGCCACCCCGGTTGGTACCGGTTGCAG

GTCGGCATCGAGCAGATAGCTTTGAGTGTTGTGCAATGGCCGGCCAATATTG

GCCCGCCCGCCGCCCTCGCGGCGAGTCCAGGTAGAGTAGGTGGTGTCTTCGG

ACGGGCCGTAAAGATCGAAGATGTGCTTGATCGTGGCTTGTTGGTAGAGGCT

GTCCACCAGCGACTGCTTCAGCGGTTCGCCGGCCAGGTTGATGATGCGCACG

CCGTCAGGGATTTGTCCGGCTCGCTGCAGGGCATTGATCGCCGATGGAACGG

TATTTATCAGGCGGACCTGTTCCCGCCCCAGCAATTCCGGCAGCTCCAGGGC

ATTGCGTGCAAGGACGATTGAGCCGCCATTGGCCAGGGTGACGAAGATTTCC

CAGACCGACAGGTCGAAGCACACCGAAGTGGAGGCCAGCACGCCCTGGATG

TCCTCACGACTATAGACTTGCTGTGACCAATGGATCAGCGCCAGCACATTGC

GATGAGCAATCGCCACACCCTTCGGCTCACCCGTA

>MG_AD21

CAGGTCGCCGGTGCGGTACAACCGTTCACCCGTAGTGGAGAACGGGTTGGG

TACATATTTTTCCGCAGTCATGGCTGCGCGCCCGAGATAGCCTCGGGTAATCC

CTGCACCTGCCAAGTGCAGTTCGGCCGCCACCCCGGTTGGTACCGGTTGCAG

GTCGGCATCGAGCAGATAGCTTTGAGTGTTGTGCAATGGCCGGCCAATATTG

GCCCGCCCGCCGCCCTCGCGGCGAGTCCAGGTAGAGTAGGTGGTGTCTTCGG

ACGGGCCGTAAAGATCGAAGATGTGCTTGATCGTGGCTTGTTGGTAGAGGCT

GTCCACCAGCGACTGCTTCAGCGGTTCGCCGGCCAGGTTGATGATGCGCACG

CCGTCAGGGATTTGTCCGGCTCGCTGCAGGGCATTGATCGCCGATGGAACGG

TATTTATCAGGCGGACCTGTTCCCGCCCCAGCAATTCCGGCGGCTCCAGGGC

ATTGCGTGCAAGGACGATTGAGCCGCCATTGGCCAGGGTGACGAAGATTTCC

CAGACCGACAGGTCGAAGCACACCGAAGTGGAGGCCAGCACGCCCTGGATG

TCCTCACGACTATAGACTTGCTGCGACCAATGGATCAGCGCCAGCACATTGC

GATGAGCAATCGCCACACCCTTCGGCTCACCCGTA

>MG_AD16

CAGGTCACCAGTGCGGTACAACCGTTCACCCGTAGTGGAGAACGGGTTGGG

TACATATTTTTCCGCAGTCATGGCTGCGCGCCCGAGATAGCCTCGGGTAATCC

CTGCACCTGCCAAGTGCAGTTCGGCCGCCACCCCGGTTGGTACCGGTTGCAG

GTCGGCATCGAGCAGATAGCTTTGAGTGTTGTGCAATGGCCGGCCAATATTG

GCCCGCCCGCCGCCCTCGCGGCGAGTCCAGGTAGAGTAGGTGGTGTCTTCGG

ACGGGCCGTAAAGATCGAAGATGTGCTTGATCGTGGCTTGTTGGTAGAGGCT

GTCCACCAGCGACTGCTTCAGCGGTTCGCCGGCCAGGTTGATGATGCGCACG

CCGTCAGGGATTTGTCCGGCTCGCTGCAGGGCATTGATCGCCGATGGAACGG

TATTTATCAGGCGGACCTGTTCCCGCCCCAGCAATTCCGGCAGCTCCAGGGC

ATTGCGTGCAAGGACGATTGAGCCGCCATTGGCCAGGGTGACGAAGATTTCC

CAGACCGACAGGTCGAAGCACACCGAAGTGGAGGCCAGCACGCCCTGGATG

TCCTCACGACTATAGACTTGCTGCGACCAATGGATCAGCGCCAGCACATTGC

GATGAGCAATCGCCACACCCTTTGGAACGCCGGTA

>MG_AD14

CAGGTCACCGGTTCGGTACAACCGTTCACCCGTAGTGGAGAACGGGTTGGG

TACATATTTTTCCGCAGTCATGGCTGCGCGCCCGAGATAGCCTCGGGTAATCC

CTGCACCTGCCAAGTGCAGTTCGGCCGCCACCCCGGTTGGTACCGGTTGCAG

GTCGGCATCGAGCAGATAGCTTTGAGCGTTGTGCAATGGCCGGCCAATATTG

GCCCGCCCGCCGCCCTCGCGGCGAGTCCAGGTAGAGTAGGTGGTGTCTTCGG

ACGGGCCGTAAAGATCGAAGATGTGCTTGATCGTGGCTTGTTGGTAGAGGCT

GTCCACCAGCGACTGCTTCAGCGGTTCGCCGGCCAGGTTGATGATGCGCACG

CCGTCAGGGATTTGTCCGGCTCGCTGCAGGGCATTGATCGCCGATGGAACGG

TATTTATCAGGCGGACCTGTTCCCGCCCCAGCAATTCCGGCGGCTCCAGGGC

ATTGCGTGCAAGGACGATTGAGCCGCCATTGGCCAGGGTGACGAAGATTTCC

CAGACCGACAGGTCGAAGCACACCGAAGTGGAGGCCAGCACGCCCTGGATG

TCCTCACGACTATAGACTTGCTGCGACCAATGGATCAGCGCCAGCACATTGC

GATGAGCAATCGCCACACCCTTAGGCACGCCGGTA

>MG_AD6

CAGGTCGCCGGTCCGATACAACCGTTCACCCGTAGTGGAGAACGGGTTGGGT

ACATATTTTTCCGCAGTCATGGCTGCGCGCCCGAGATAGCCTCGGGTAATCCC

TGCACCTGCCAAGTGCAGTTCGGCCGCCACCCCGGTTGGTACCGGTTGCAGG

TCGGCATCGAGCAGATAGCTCTGAGTGTTGTGCAATGGCCGGCCAATATTGG

CCCGCCCGCCGCCCTCGCGGCGAGTCCAGGTAGAGTAGGTGGTGTCTTCGGA

CGGGCCGTAAAGATCGAAGATGTGCTTGATCGTGGCTTGTTGGTAGAGGCTG

TCCACCAGCGACTGCTTCAGCGGTTCGCCGGCCAGGTTGATGATGCGCACGC

CGTCAGGGATTTGTCCGGCTCGCTGCAGGGCATTGATCGCCGATGGAACGGT

ATTTATCAGGCGGACCTGTTCCCGCCCCAGCAATTCCGGCAGCTCCAGGGCA

TTGCGTGCAAGGACGATTGAGCCGCCATTGGCCAGGGTGACGAAGATTTCCC

AGACCGACAGGTCGAAGCACACCGAAGTGGAGGCCAGCACGCCCTGGATGT

CCTCACGACTATAGACTTGCTGCGACCAATGGATCAGCGCCAGCACATTGCG

ATGAGCAATCACCACACCCCTTAGGCACGCCCGTA
